# The p38-MITOGEN-ACTIVATED PROTEIN KINASE Signaling Pathway Is Involved in *Leonurus artemisia* Extract-Induced Inhibition of the Proliferation of Human Bladder Cancer BFTC-905 Cells via G1/G0 Arrest and Causes Apoptosis In Vitro

**DOI:** 10.3390/ph16101338

**Published:** 2023-09-22

**Authors:** Jian-Hui Lin, Chein-Hui Hung, Yun-Ching Huang, Chih-Shou Chen, Dong-Ru Ho

**Affiliations:** 1 Division of Urology, Department of Surgery, Chang Gung Memorial Hospital, Chiayi 613, Taiwan; b9005026@gmail.com (J.-H.L.); dr5326@cgmh.org.tw (Y.-C.H.);; 2 Graduate Institute of Clinical Medical Sciences, College of Medicine, Chang Gung University, Taoyuan 333, Taiwan; hungc01@mail.cgu.edu.tw; 3 School of Medicine, National Tsing Hua University, Hsinchu 300044, Taiwan

**Keywords:** *Leonurus artemisia* extract, Chinese motherwort, Scientific Chinese Medicine extract, bladder cancer, BFTC-905 cells, reactive oxygen species, mitochondrial pathway-dependent apoptosis, p38-mitogen-activated protein kinase signaling pathway, G1/G0 arrest

## Abstract

Bladder cancer is a urothelial malignancy. Bladder cancer starts in the urothelial cells lining the inside of the bladder. The 5-year recurrence rate for bladder cancer ranges from 31% to 78%, and the progression rate is approximately 45%. To treat bladder cancer, intravesical drug therapy is often used. *Leonurus artemisia* extract (LaE) was obtained from medicinal samples of Chinese motherwort Scientific Chinese Medicine; *L. artemisia* has various biological effects. This study investigated the impact of LaE on human bladder cancer cells (the BFTC-905 cell line) and the molecular mechanism underlying apoptosis resulting from the activation of cell signal transduction pathways in bladder cancer cells. A cell counting kit-8 (CCK-8) assay was used to determine the effect of LaE on cell growth. The effect of LaE on migration ability was observed using a wound healing assay. The effects of LaE on the cell cycle, reactive oxygen species production, and apoptosis were investigated. Western blot analysis detected apoptosis-related and mitogen-activated protein kinase signaling pathway-related protein concentrations. At non-toxic concentrations, LaE inhibited the proliferation of BFTC-905 cells in a concentration-dependent manner, and the half-maximal inhibitory concentration (IC50) was 24.08172 µg/µL. LaE impaired the migration ability of BFTC-905 cells. LaE arrested the cell cycle in the G1 and G0 phases, increased reactive oxygen species production, and induced apoptosis. LaE increased Bax and p-ERK concentrations and decreased Bcl-2, cleaved caspase-3, and p-p38 concentrations. No differences in PARP, C-PARP, vimentin, e-cadherin, p-JNK, or TNF-alpha concentrations were observed. These results suggest that LaE inhibits the proliferation of human bladder cancer cells. Moreover, the mitogen-activated protein kinase signaling pathway is involved in the inhibition of the proliferation of BFTC-905 cells.

## 1. Introduction

Bladder cancer is a significant global health concern and the ninth most common cancer worldwide [1,2]. Non-muscle-invasive bladder cancer accounts for approximately 75% of cases, and muscle-invasive bladder cancer accounts for 25% [3]. Metastasis is observed in 10–15% of muscle-invasive bladder cancer cases. The therapeutic strategy for bladder cancer depends on the subtype, which is identified through histological examination. For non-muscle-invasive bladder cancer, the primary approach is transurethral resection combined with intravesical chemotherapy or immunotherapy [4]. The recurrence and progression rates in non-muscle-invasive bladder cancer are high, resulting in poor patient outcomes. The 5-year recurrence rate ranges from 31% to 78%, and progression rates can reach 45% [5]. To minimize recurrence and progression, intravesical drug therapy is often used. Among the agents used to treat non-muscle-invasive bladder cancer, the Bacillus Calmette–Guérin vaccine is the most effective; however, its side effects include increased urinary frequency and urgency, cystitis, and hematuria [6]. Therefore, new intravesical agents are needed that are both effective and safe.

*Leonurus japonicus* Houtt, commonly called Chinese motherwort, is a traditional herbal medicine used to treat dysmenorrhea, amenorrhea, and postpartum hemorrhage [7]. In China, L. japonicus is known as *Leonurus artemisia*. The herb has several physiological, pharmacological, and biological effects, including cardioprotective, antioxidant, neuroprotective, and anticancer effects [8]. Chinese motherwort extract can induce apoptosis in several tumor cell lines in a concentration-dependent and time-dependent manner, and the mechanism of apoptosis involves the mitochondria [9]. Aqueous ethanol-extracted Chinese motherwort inhibits the proliferation of breast cancer cells through mechanisms involving cytotoxicity and cell cycle arrest [10] and inhibits the proliferation of lung cancer cells [11]. In one study, methanolic extract of Chinese motherwort induced apoptosis in the Huh-7 and HSC-T6 human hepatic carcinoma cell lines, possibly through a mechanism involving a mitochondrial signaling pathway [12]. Thus, Chinese motherwort has anticancer effects on breast, lung, and liver cancer cell lines.

Approximately 140 chemical compounds are discerned from Leonurus japonicus, with primary components such as alkaloids, diterpenes, and flavones. Among these, leonurine and stachydrine have been extensively studied [13]. While leonurine has emerged as a key active compound in many studies [14], its application in treating bladder cancer is not economically viable due to its elevated cost. A more cost-effective and straightforward alternative might be the use of a motherwort-based Chinese medicinal extract.

No studies have investigated the effects of Chinese motherwort extract on bladder cancer. Experiments investigating the potential effects and safe concentrations of Chinese motherwort are warranted. The present study investigated the effects of a Chinese motherwort on a human bladder cancer cell line. The results provide new insights into the anticancer effects of Chinese motherwort. They may be helpful for the potential application of Chinese motherwort as a natural treatment option for bladder cancer.

## 2. Results

### 2.1. Leonurus artemisia Extract Inhibited the Proliferation of Human Bladder Cancer Cells

**Figure 1:**
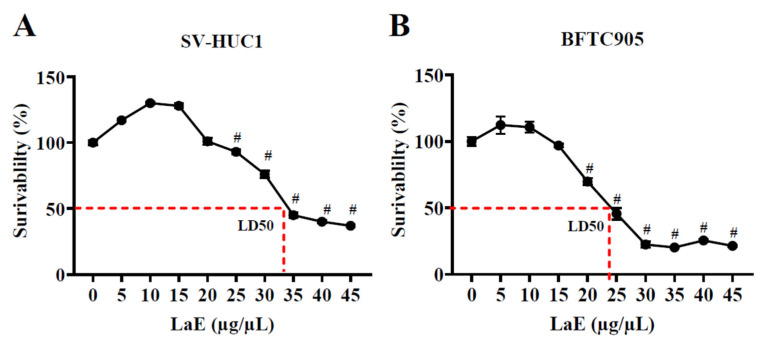
Effect of *L. artemisia* extract (LaE) on human bladder cancer and normal urothelial cell proliferation. Cells were treated with LaE for 24 h; cell viability was detected using CCK-8 assay. Growth curves of (**A**) SV-HUC-1 (normal) cells and (**B**) BFTC-905 (cancer) cells. (# *p* < 0.0001 compared to control).

*L. artemisia* extract (LaE) inhibited the proliferation of BFTC-905 human bladder cancer cells in a concentration-dependent manner. Its IC50 was 24.08172 µg/µL (Figure 1B). At low concentrations, it was non-toxic. After 24 h of treatment with LaE (25 µg/µL), cell viability of cancer (Figure 1B) and normal cells (Figure 1A) was 45.55% and 93.18%, respectively.

### 2.2. LaE Inhibited Migration Ability of Human Bladder Cancer Cells

**Figure 2:**
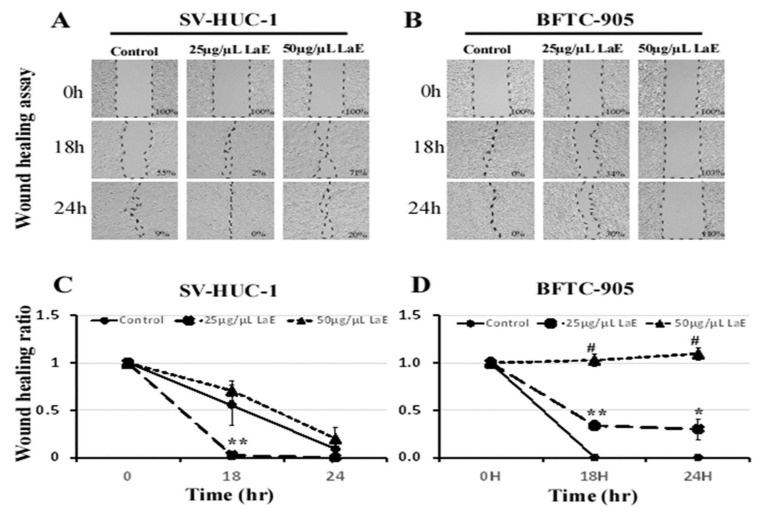
The migration ability of SV-HUC-1 (**A**) and BFTC-905 cells (**B**) was evaluated using a wound healing assay after treatment with LaE (25 and 50 µg/µL) for 0, 18, and 24 h. The areas of the wounds were calculated, and statistical analysis revealed significant differences (* *p* < 0.05; ** *p* < 0.005; # *p* < 0.0001) compared to the control group (**C**,**D**).

Human bladder cancer cells and normal bladder urothelial (SV-HUC-1) cells were treated with LaE (25 and 50 µg/µL) for 18 or 24 h. Migration ability was evaluated using a wound healing assay/migration assay. LaE significantly inhibited the migration ability of the human bladder cancer cells (Figure 2B), but its effect on the normal bladder urothelial cells was non-significant (Figure 2A). The areas of the wounds were calculated, and statistical analysis revealed significant differences (* *p* < 0.05; ** *p* < 0.005; # *p* < 0.0001) compared to the control group (Figure 2C,D).

### 2.3. LaE Induced Apoptosis in Human Bladder Cancer Cells

**Figure 3:**
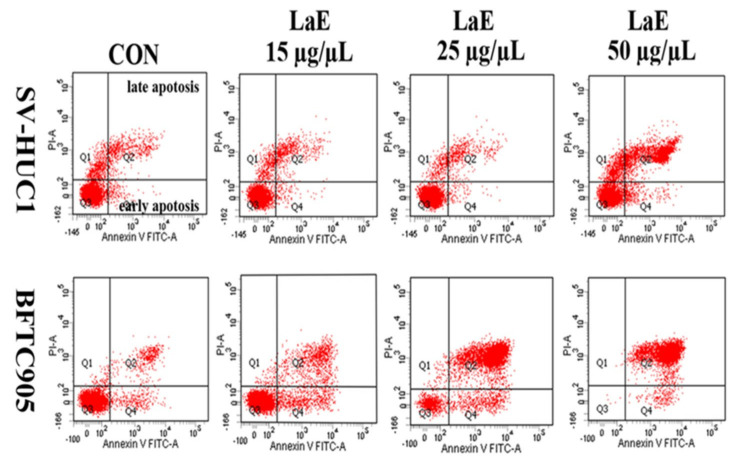
Effect of LaE on apoptosis in BFTC-905 and SV-HUC-1 cells. After being treated with LaE for 24 h, BFTC-905, and SV-HUC-1 cells were stained with annexin V-fluorescein isothiocyanate and propidium iodide and analyzed using flow cytometry. Effects of LaE (0, 15, 25, and 50 µg/µL) on apoptosis were analyzed.

**Figure 4:**
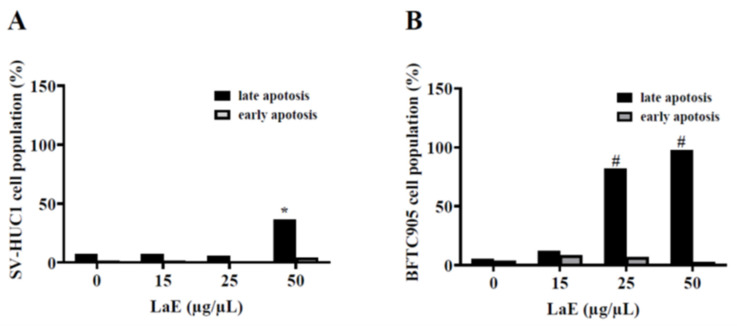
Apoptosis rate was measured in SV-HUC-1 (**A**) and BFTC-905 cells (**B**) treated with LaE at 0, 15, 25, and 50 µg/µL. Cancer cells showed more severe late apoptosis than normal cells (* *p* < 0.05, # *p* < 0.0001 compared to control).

Human bladder cancer cells were treated with LaE for 24 h. Apoptosis was measured using flow cytometry. The LaE-treated cells were stained with annexin V-fluorescein isothiocyanate and propidium iodide. LaE induced apoptosis in a concentration-dependent manner. The apoptosis rates of the bladder cancer cells treated with 0, 15, 25, and 50 µg/µL LaE were 8.6%, 20.3%, 88.5%, and 99.8%, respectively (Figure 3). The apoptosis rates of the SV-HUC-1 cells treated with 0, 15, 25, and 50 µg/µL LaE were 8.8%, 8.4%, 6.4%, and 40.3%, respectively (Figure 3). Both cancer and normal cell lines were biased toward late apoptosis (Figure 4A,B). Bladder cancer cells showed more severe late apoptosis than normal cells (* *p* < 0.05, # *p* < 0.0001 compared to control).

### 2.4. LaE Induced Apoptosis via Mitogen-Activated Protein Kinase Cascade Signaling

**Figure 5:**
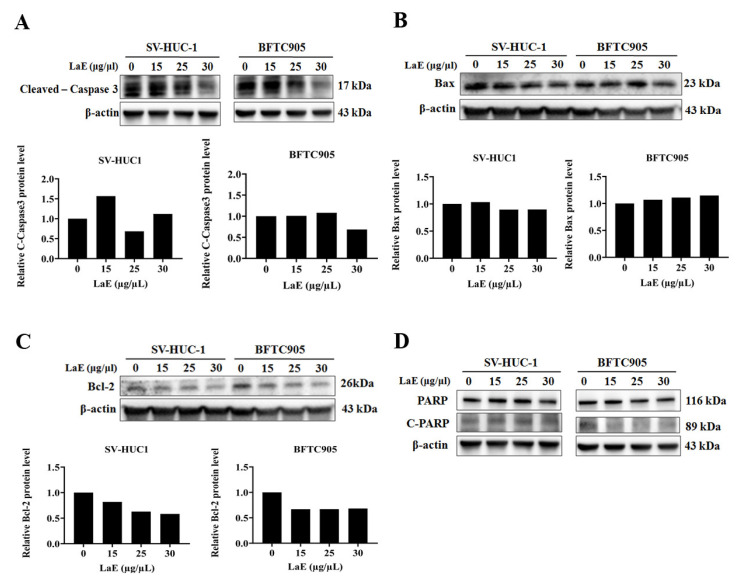
Western blot analysis detected concentrations of cleaved caspase-3 (**A**), Bax (**B**), Bcl-2 (**C**), and PARP/C-PARP (**D**). In BFTC-905 cells, cleaved caspase-3 concentrations were not elevated, and no changes in PARP or C-PARP concentrations were observed (**A**,**D**). Bax was slightly elevated in the BFTC-905 group (**B**), and Bcl-2 decreased in both groups (**C**).

Western blot analysis was utilized to measure concentrations of apoptosis-linked proteins. LaE slightly diminished the concentrations of cleaved caspase-3 (Figure 5A), poly (ADP-ribosyl) polymerase (PARP) and cleaved-PARP (Figure 5D). However, this did not conclusively establish that LaE suppressed BFTC-905 cell growth via apoptosis induction. This study further probed the concentrations of Bax, Bcl-2, p-ERK, and p-p38, which are implicated in the mitochondrial and mitogen-activated protein kinase (MAPK) signaling pathways using Western blot analysis.

The MAPK signaling pathway comprises three protein subfamilies: ERK, p38, and JNK [15]. Various stimuli activate and regulate physiological processes such as cell growth and death, and they play essential roles in apoptosis. To investigate the effect of LaE on the MAPK signaling pathway in BFTC-905 cells, the concentrations of MAPK signaling pathway-related proteins were detected using Western blot analysis.

**Figure 6:**
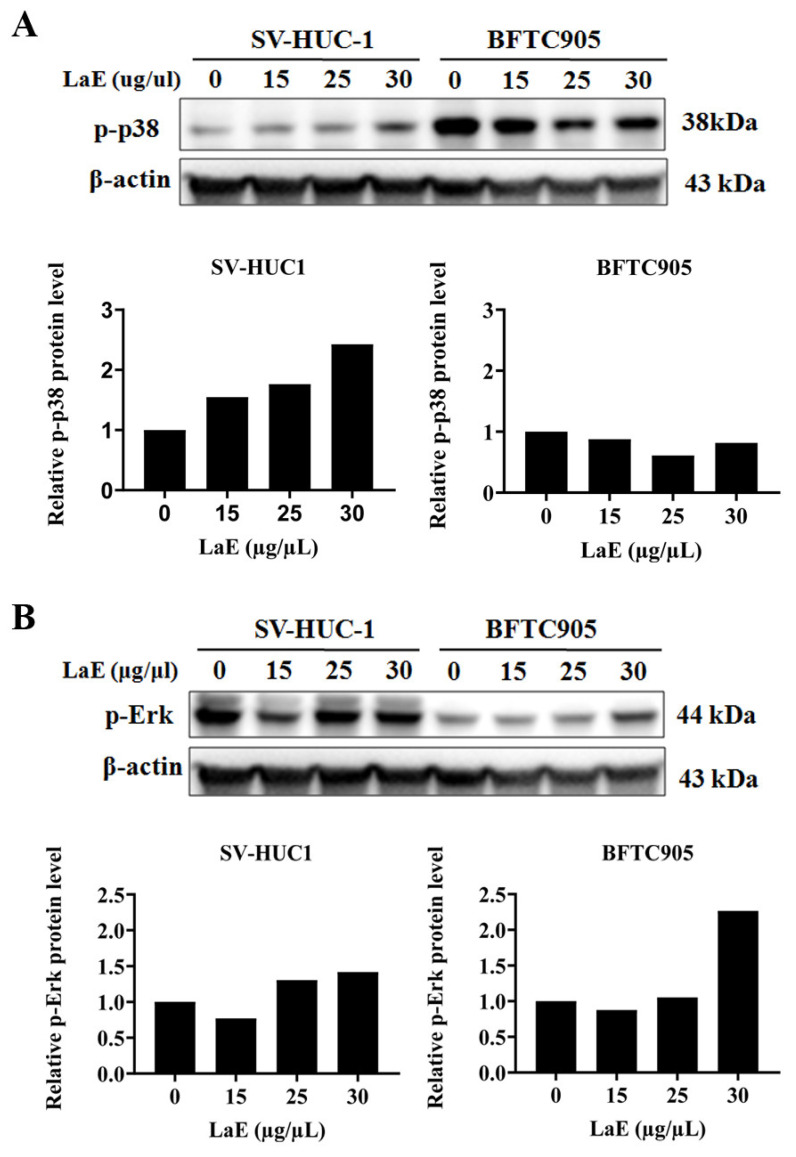
Western blot analysis detected p-p38 (**A**) and p-ERK (**B**) concentrations. A significant difference in p-p38 concentration was observed between SV-HUC-1 and BFTC-905 cells. The levels were decreased in BFTC 905 cells and increased in SV-HUC-1 cells. The treatment of LaE (30 µg/µL) showed a significant increase in p-ERK concentrations in BFTC 905 cells.

LaE significantly decreased the concentrations of Bcl-2 (Figure 5C) and slightly increased those of the proapoptotic protein Bax in the BFTC-905 cells (Figure 5B). Mitochondrial pathway-dependent apoptosis may have been induced by LaE. LaE significantly reduced concentrations of p-p38 (Figure 6A) in BFTC 905 cell group. A significant difference in p-p38 concentration was observed between SV-HUC-1 and BFTC-905 cells. The levels were decreased in BFTC 905 cells but increased in SV-HUC-1 cells. Elevated concentrations of p-ERK were observed in both bladder cancer and normal bladder cells (Figure 6B). The treatment of LaE (30 µg/µL) showed a significant increase in p-ERK concentrations in BFTC 905 cells (Figure 6B).

**Figure 7:**
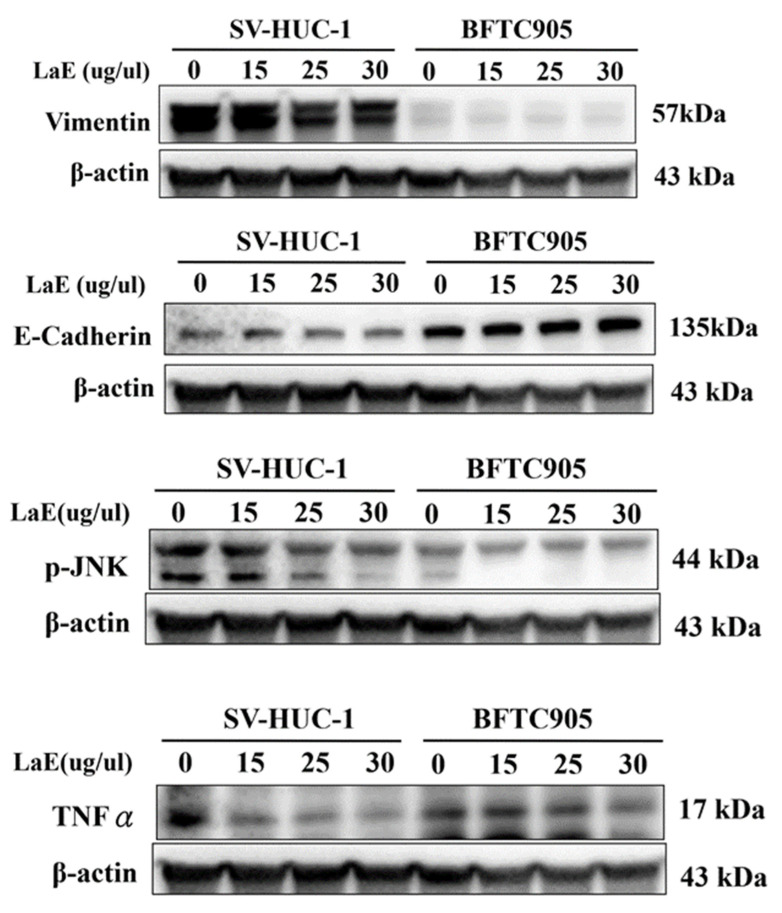
Western blot analysis detected vimentin, E-cadherin, p-JNK, and TNF-alpha concentrations. No noticeable changes in concentrations of these proteins were observed in either SV-HUC-1 or BFTC-905 cells.

These findings suggest that the LaE-induced inhibition of bladder cancer cell proliferation involves the MAPK signaling pathway. We did not observe any significant changes in the concentrations of vimentin, E-cadherin, p-JNK, or TNF-alpha (Figure 7).

### 2.5. LaE Increased Reactive Oxygen Species Production

**Figure 8:**
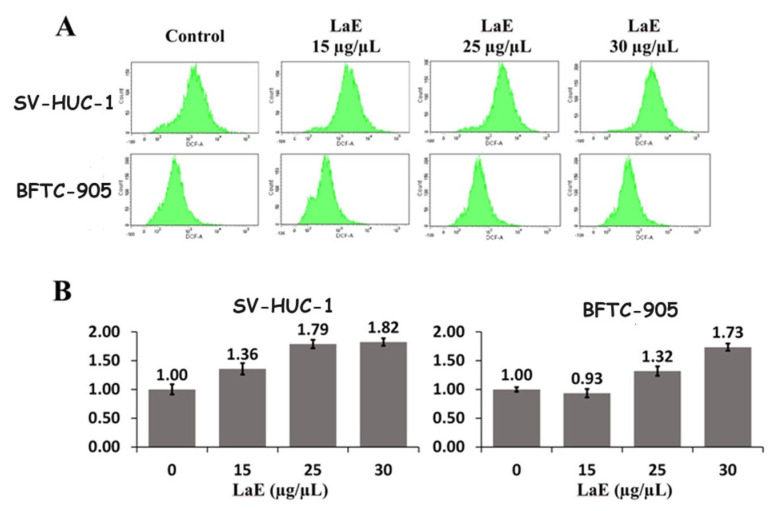
SV-HUC-1 and BFTC 905 cells were treated with 15, 25, and 30 µg/µL LaE for 24 h, stained with DCF-A and propidium iodide, and analyzed using flow cytometry to measure ROS levels (**A**). The ROS levels of the treated cells were compared with the control group (**B**).

To investigate whether oxidative stress was involved in the LaE-induced apoptosis in bladder cancer cells, a reactive oxygen species (ROS) assay was performed on LaE-treated SV-HUC-1 and BFTC-905 cells. The cells were stained with 2′,7′- dichlorofluorescein diacetate (DCF-DA)/propidium iodide (PI). The SV-HUC-1 and BFTC-905 cells were treated with 15, 25, or 30 µg/µL of LaE for 24 h (Figure 8A).

The ROS values produced in SV-HUC-1 and BFTC 905 cells treated with LaE at 0, 15, 25, and 30 µg/µL are presented in Figure 8B. SV-HUC-1 cells produced 2341, 3174, 4188, and 4269, while BFTC 905 cells produced 545, 509, 718, and 945 ROS, respectively. Both groups showed an increase in ROS production. However, the SV-HUC-1 group had higher ROS concentrations than the BFTC-905 group (Figure 8).

### 2.6. LaE Induced Cell Cycle Arrest in G1 and G0 Phases

**Figure 9:**
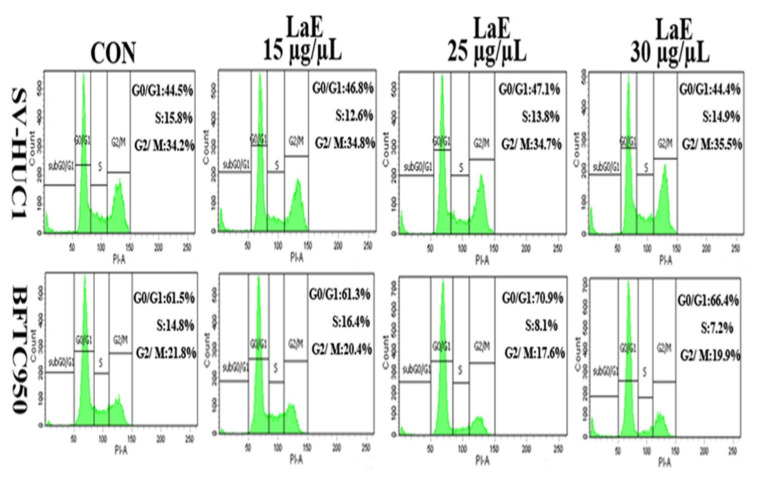
After treating SV-HUC-1 and BFTC-905 cells with varying concentrations of LaE (0, 15, 25, and 30 µg/µL) for 24 h, SV-HUC-1 and BFTC-905 cells stained with propidium iodide were collected using flow cytometry to analyze cell cycle distribution.

**Figure 10:**
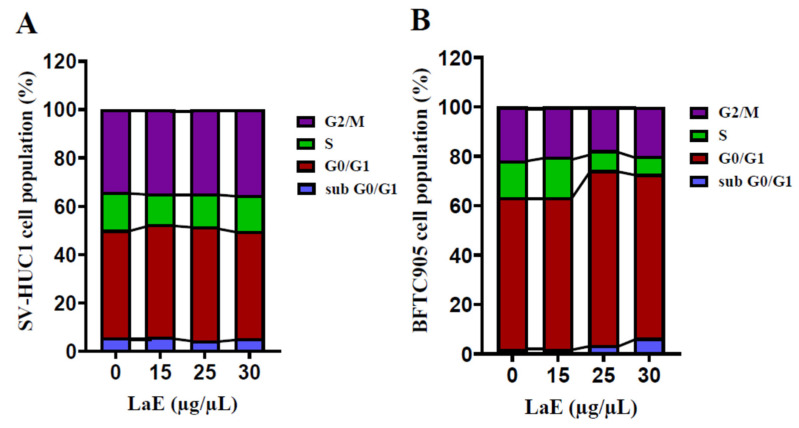
The proportion of cell cycle groups in SV-HUC-1 (**A**) and BFTC-905 cells (**B**). Under increased concentrations of LaE, the proportion of cells in the synthesis phase decreased significantly for BFTC-905 cells (**B**). In the BFTC-905 cells treated with 25 µg/µL LaE group, the proportion of cells in the synthesis phase decreased from 14.8% to 8.1% compared to the control group (0 µg/µL LaE), and the difference was statistically significant.

In investigating the inhibitory role of LaE on cell growth, we analyzed the cell cycle distributions of SV-HUC-1, and BFTC-905 cells post-treatment with various concentrations of LaE, utilizing propidium iodide staining and flow cytometry. Remarkably, BFTC-905 cells demonstrated a significant alteration in their cell cycle dynamics in response to LaE treatment. As depicted in Figure 9, an elevated proportion of cells were found in the G1 and G0 phases with the progression of LaE concentration from 0 to 30 µg/µL, highlighting a concentration-dependent increase in cells in the arrest phase. Concurrently, a marked reduction in the synthesis phase population was observed, delineating from 14.8% in the control to 8.1% and 7.2% following treatment with 25 and 30 µg/µL of LaE, respectively. As substantiated in Figure 10, this reduction was statistically significant, illustrating a conspicuous inverse relationship with the LaE concentration. In contrast, SV-HUC-1 cells remained largely unaffected, showcasing no substantial deviations in the cell cycle phases across varied LaE concentrations, as corroborated by Figure 10. Synthesizing these findings, it becomes apparent that LaE instigates a cell cycle arrest predominantly in the G1 and G0 phases while triggering late apoptosis in BFTC-905 cells. The unaffected SV-HUC-1 cell cycle articulates a selective action of LaE, laying a foundation for further explorations into the mechanistic pathways engaged by LaE in inducing cell cycle arrests and apoptosis. This avenue of investigation opens up promising directions for leveraging LaE’s selective inhibitory effects in therapeutic applications, harboring potential implications in targeted cancer therapies.

## 3. Discussion

The European Medicines Agency has described the composition of *L. artemisia*. It contains furan diterpenes (labdanes), alkaloids (notably stachydrine), sterols, iridoids, flavonoids, ursolic acid, and minerals [16]. LaE is derived from the previously extracted Motherwort Scientific Chinese Medicine. Performing a re-extraction during experiments could modify its original composition. The objective of this study was to deter cancer recurrence by infusing the bladder with this scientific Chinese medicine extract. While leonurine is recognized as the primary active compound in motherwort, its high cost makes it impractical for standalone bladder cancer treatment. Hence, considering a motherwort extract as a treatment is both economical and more direct.

Chinese motherwort is a traditional herbal medicine for treating dysmenorrhea, amenorrhea, and postpartum hemorrhage [5]. It has various physiological, pharmacological, and biological effects, including cardioprotective, antioxidant, neuroprotective, and anticancer effects [6]. The anticancer effects of Chinese motherwort on breast, lung, and liver cancer cells may involve a ROS-mediated mitochondrial signaling pathway and cell cycle arrest [7,8,9,10].

In the present study, LaE inhibited the proliferation of human bladder cancer cells in a concentration-dependent manner, with an IC50 value of 24.08172 µg/µL. LaE did not have a toxic effect on normal human bladder urothelial cells within the effective concentration range. Further exploring the mechanism of the antiproliferative effect of LaE on BFTC-905 cells, this study found that LaE arrested the cell cycle in the G1 and G0 phases and decreased the proportion of BFTC-905 cells in the synthesis phase. Thus, LaE may arrest the cell cycle and induce apoptosis in human bladder cancer.

The apoptosis rates of the human bladder cancer cells treated with 0, 15, 25, and 50 µg/µL LaE for 24 h were 8.6%, 20.3%, 88.5%, and 99.8%, respectively, and the cells were biased toward late apoptosis. Furthermore, according to the Western blot analysis, LaE did not elevate cleaved PARP or Caspase-3 levels. Therefore, we hypothesized that LaE does not induce apoptosis through the typical pathway. Studies show that the concentration of Bax and Bcl-2 can predict bladder cancer treatment outcomes [17,18,19]. In our research, LaE reduced Bcl-2 concentrations and slightly increased proapoptotic protein Bax in BFTC-905 cells.

Thus, mitochondrial pathway-dependent apoptosis involves decreased concentrations of Bcl-2 induced by LaE. However, the ROS assay in this study revealed ROS overproduction in both cancer and normal bladder cells, with higher concentrations of ROS in the SV-HUC-1 cells. ROS accumulation results in oxidative stress-induced apoptosis in bladder cancer cells, and some bladder cell line apoptosis studies revealed that the generation of ROS and DNA damage may be the significant mechanism [20,21]. The apoptosis of the BFTC-905 cells did not need high oxidative stress and caused more considerable late apoptosis change.

Therefore, further investigation of the MAPK pathway is necessary. Western blot analysis indicated that LaE significantly reduced levels of p-p38 in human bladder cancer cells (BFTC 905 cells) compared to SV-HUC-1 cells. SV-HUC-1 cells treated with LaE exhibited increased levels of p-p38. Elevated concentrations of p-ERK were observed in both normal bladder and cancer cells. No noticeable changes in vimentin, E-cadherin, p-JNK, or TNF-alpha concentrations were observed. A wound healing assay was conducted to evaluate the migration ability of bladder cancer cells compared to normal bladder urothelial cells. LaE inhibited the migration of the cancer cells but not the normal cells.

The activation of p38-mitogen-activated protein kinase (p38 MAPK) initiates a signaling cascade that significantly contributes to diverse cellular processes [22]. P38 MAPK regulates myoblast differentiation by affecting transcription factor activity, chromatin remodeling, and the turnover of specific mRNAs encoding muscle differentiation regulators [23]. Moreover, there is ample evidence linking p38 MAPK to apoptosis. Activation of p38 MAPK often occurs simultaneously with apoptosis induced by various agents such as the withdrawal of nerve growth factor (NGF) or the ligation of the Fas receptor [24].

The involvement of p38 MAPK in apoptosis is well-established and has been extensively studied. Additionally, several reports suggest that p38 MAPK is implicated in regulating the G1 and G2/M phases of the cell cycle [25]. The G1 phase involves cell growth and preparation for DNA replication. Multiple studies have indicated that p38 MAPK plays a role in modulating the progression of cells through these specific cell cycle phases [26,27,28]. This p38-MAPK signaling pathway is also known for its role in chemotactic cell migration, primarily associated with its impact on cytoskeleton changes [29].

Our study analyzed the cell cycle distribution of LaE-treated BFTC-905 cells. The proportions of BFTC-905 cells in the G1 and G0 phases were 61.5%, 61.3%, 70.9%, and 66.4% after treatment with 0, 15, 25, and 30 µg/µL LaE, respectively. Additionally, the proportion of BFTC-905 cells in the synthesis phase decreased significantly. The proportion of BFTC-905 cells in the synthesis phase was significantly lower after 25 µg/µL LaE treatment than after no treatment (8.1% vs. 14.8%, respectively). Activation of the p38-MAPK signaling pathway by LaE results in cell cycle arrest in the G1 and G0 phases and cell growth inhibition during the synthesis phase. ROS-mediated mitochondrial apoptosis pathways were activated, accompanied by increased reactive oxygen species production due to LaE. Our study found that the treatment with LaE resulted in a notable increase in bladder cancer cell migration inhibition.

These findings indicate that the p38-MAPK signaling pathway is involved in the LaE-induced inhibition of the proliferation of bladder cancer cells BFTC 905 Cells via cell cycle arrest in the G1 and G0 phases and causes apoptosis in vitro. The results provide new insights into the anticancer effects of Chinese motherwort. They may be helpful for the potential application of Chinese motherwort as a natural treatment option for bladder cancer. Additionally, research regarding the effect of LaE on bladder cancer treatment in animal models will be carried out. Our study is limited by using a single bladder cancer cell line (BFTC 905), which might not capture the full spectrum of LaE’s effects on bladder cancer. Secondly, variations in LaE composition due to the re-extraction process could have implications for the reproducibility and interpretation of our results. As LaE is derived from Motherwort Scientific Chinese Medicine—a substance already extracted—our experimental extraction could have altered its native composition. Furthermore, the absence of a comparison between the LaE and BCG studies is notable. Therefore, to confirm these findings, more experiments may be required. Additional research is currently being conducted.

## 4. Materials and Methods

### 4.1. Materials

Chinese motherwort (*L. artemisia*) (Scientific Chinese Medicine) was obtained from the Chiayi Chang Gung Memorial Hospital. Roswell Park Memorial Institute (RPMI)-1640 basal medium supplemented with fetal bovine serum (FBS) was obtained from Gibco (Grand Island, NE, USA). A cell counting kit-8 (CCK-8) was obtained from Dojindo (Kumamoto Prefecture, Japan). The wound healing assay, annexin V, fluorescein isothiocyanate, propidium iodide, cell cycle and apoptosis analysis kit, and lysis buffer for the radioimmunoprecipitation assay were obtained from Blossom Biotechnologies (Taipei, Taiwan). Phosphatase inhibitor cocktail tablets (PhosSTOP) were received from Roche (Basel, Switzerland). Bovine serum albumin fraction V and ultrasensitive enhanced chemiluminescence substrate were obtained from Biosharp (Hefei, China). A bicinchoninic acid protein assay kit was obtained from Vazyme (Nanjing, China). Antibodies against cleaved caspase-3, PARP, cleaved PARP, Bax, Bcl-2, p-p38, p-JNK, p-ERK, and GAPDH and horseradish peroxidase-conjugated goat antirabbit antibodies were purchased from CST (Boston, MA, USA). All other chemicals were of analytical grade.

### 4.2. Cell Culture

Human bladder cancer cells (BFTC-905 cells) and normal bladder urothelial cells (SV-HUC-1 cells) were purchased from the Food Industry Research and Development Institute (Hsinchu, Taiwan) and cryopreserved in a liquid nitrogen tank. By the culture method recommended by the American Type Culture Collection, the cells were cultured in RPMI-1640 medium containing 10% FBS at 37 °C with 5% CO_2_. The cells were used in the subsequent experiments once they had reached logarithmic growth.

### 4.3. Extraction of Chinese Motherwort (L. artemisia)

Motherwort (Scientific Chinese Medicine) (1 g) was mixed with 10 mL of F12 medium 7% FBS / RPMI-1640 medium 15% FBS. The LaE was collected by filtering the mixture left to rotate overnight in a cold room with a mixer through a 0.22-μm filter.

### 4.4. Cell Proliferation and Cytotoxicity Assay

BFTC-905 and SV-HUC-1 cells were inoculated separately and at the same density into 96-well cell culture plates overnight. Subsequently, LaE was added at concentrations of 0, 5, 10, 15, 20, 25, 30, 35, 40, or 45 µg/µL, and the plates were incubated for 24 h. CCK-8 solution was added to each well, and the cells were incubated for another 4 h. Absorbance at 450 nm was measured using a multimode plate reader (PerkinElmer VICTOR Nivo, Waltham, MA, USA). GraphPad Prism 9.0 software drew the BFTC-905 and SV-HUC-1 cells’ growth curves and calculated IC50 values.

### 4.5. Wound Healing Assay

The 3 × 10^4^ SV-HUC-1 and BFTC-905 cell suspensions are added to the well with the insert in place. They are then incubated for 24 h until a monolayer forms. The insert is then removed to generate a 0.9 mm wound field. Control and treatment groups (25 and 50 µg/µL LaE for 24 h) were established. Cell migration was observed at 0, 18, and 24 h. Wound closure was monitored with a light microscope and imaging software (image-pro plus 6.0 software), and wound areas were calculated.

### 4.6. Cell Apoptosis Assay

After treatment with LaE for 24 h, SV-HUC-1, and BFTC-905 cells were collected through centrifugation and resuspended in a staining buffer containing annexin V-fluorescein isothiocyanate and propidium iodide. After staining in the dark for 20 min, a flow cytometer was used to detect and analyze the apoptosis-inducing effect of LaE.

### 4.7. Western Blot Analysis

After being treated with LaE for 24 h, SV-HUC-1 and BFTC-905 cells were lysed in radioimmunoprecipitation assay lysis buffer containing phosphatase and protease inhibitor, and the lysates were centrifuged at 13,000 rpm for 20 min at 4 °C. Protein concentrations were determined using the bicinchoninic acid method. Protein mass, volume, and concentration were then adjusted to equal levels, and 50 µg of total protein was separated into proteins of different molecular weights through 12% sodium dodecyl sulfate-polyacrylamide gel electrophoresis. The proteins were then transferred to polyvinylidene fluoride membranes via wet electrotransfer at 250 mA. The membranes were blocked for 1 h with 5% bovine serum albumin at 37 °C and incubated overnight at 4 °C with the respective primary antibodies, followed by incubation with the secondary antibody conjugated to horseradish peroxidase for 2 h. The blots were visualized using an ultrasensitive enhanced chemiluminescence substrate kit (ChemiDocXRS; Bio-Rad, Hercules, CA, USA) and Quantity One image analysis software. Integrated optical density was analyzed in ImageJ. GAPDH was used as the internal reference protein. The combined optical density ratio of the target protein to the inner reference protein was considered to reflect the relative concentration of the target protein.

### 4.8. ROS Assay

The effect of LaE on ROS production in SV-HUC-1 and BFTC-905 cells treated with 0, 15, 25, or 30 µg/µL LaE for 24 h was investigated. The medium of 2′,7′-dichlorofluorescin diacetate (DCF-DA) was prepared. The DCF-DA medium contained 10 mL of 2% FBS and 10 µL of 1 mM DCF (DCF stock, 10 mM). The working concentration was 1 μM. The original medium was replaced with 1 mL of DCF-DA medium, and the cells were incubated at 37 °C for 30 min. An H_2_O_2_ group was prepared (1000× dilution). The medium was replaced with 1 mL of trypsin. Subsequently, the cells were washed using 1 mL of FBS in a 15 mL tube centrifuged at 1500 rpm for 5 min. The cells were then washed using 4 mL of phosphate-buffered solution (2% FBS) in a 15 mL tube centrifuged at 1500 rpm for 5 min. This procedure was repeated twice. The supernatant was removed, and 300 µL of propidium iodide was added. After staining with DCF-DA and propidium iodide, a flow cytometer was used to detect and analyze the proportions of fluorescent SV-HUC-1 and BFTC-905 cells.

### 4.9. Cell Cycle Assay

After being treated with 0, 15, 25, or 30 µg/µL LaE for 24 h, the SV-HUC-1 and BFTC-905 cells were collected by centrifugation. The cell precipitates were resuspended with 300 µL of precooled phosphate-buffered solution, added drop by drop to 700 µL of precooled anhydrous ethanol on a vortex mixer (Digital IR Vortex Mixer TX4, Milano, Italy), and then fixed at 4 °C overnight. After the ethanol was removed by centrifugation, the cells were resuspended with a staining solution containing staining buffer, propidium iodide, and RNase-A according to the kit instructions. After staining in the dark for 30 min, the cells were collected, and a flow cytometer (ACEA Biosciences NovoCyte Quanteon, San Diego, CA, USA) was used to analyze the cell cycle distribution.

### 4.10. Statistical Analysis

Data were analyzed in GraphPad Prism 9.0. Each group of experiments was independently performed three times. Results are expressed as the mean ± standard deviation. Differences between groups were tested using a one-way analysis of variance and the Dunnett t-test. Statistical significance was indicated by *p* < 0.05.

## 5. Conclusions

Chinese motherwort (*L. artemisia*) has been extensively researched for its biological effects. Our findings indicate that LaE inhibits the proliferation of human bladder cancer cells (i.e., the BFTC-905 cell line) in vitro and induces apoptosis by arresting the cell cycle in the G1 and G0 phases through activating the p38-MAPK signaling pathway. Additionally, LaE reduces the concentration of cells in the synthesis phase, triggers ROS-mediated mitochondrial apoptosis pathways, and inhibits migration, revealing its potential as a novel agent for bladder cancer treatment. Additional research will be carried out to study the influence of LaE in tackling bladder cancer in animal models.

## Data Availability

Data is contained within the article.
